# A microfluidic organ-on-a-chip: into the next decade of bone tissue engineering applied in dentistry

**DOI:** 10.2144/fsoa-2023-0061

**Published:** 2023-09-08

**Authors:** Muhammad Hidayat Syahruddin, Rahmi Anggraeni, Ika Dewi Ana

**Affiliations:** 1 Postgraduate Student, Dental Science Doctoral Study Program, Faculty of Dentistry, Universitas Gadjah Mada, Yogyakarta, 55281, Indonesia; 2 Research Center for Preclinical & Clinical Medicine, National Research & Innovation Agency of the Republic of Indonesia, Cibinong Science Center, Bogor, 16915, Indonesia; 3 Department of Dental Biomedical Sciences, Faculty of Dentistry, Universitas Gadjah Mada, Yogyakarta, 55281, Indonesia; 4 Research Collaboration Center for Biomedical Scaffolds, National Research & Innovation Agency (BRIN) – Universitas Gadjah Mada (UGM), Yogyakarta, 55281, Indonesia

**Keywords:** alveolar bone, Bone disease modeling, dentistry, organ-on-a-chip, periodontitis, tissue engineering

## Abstract

A comprehensive understanding of the complex physiological and pathological processes associated with alveolar bones, their responses to different therapeutics strategies, and cell interactions with biomaterial becomes necessary in precisely treating patients with severe progressive periodontitis, as a bone-related issue in dentistry. However, existing monolayer cell culture or pre-clinical models have been unable to mimic the complex physiological, pathological and regeneration processes in the bone microenvironment in response to different therapeutic strategies. In this point, ‘organ-on-a-chip’ (OOAC) technology, specifically ‘alveolar-bone-on-a-chip’, is expected to resolve the problems by better imitating infection site microenvironment and microphysiology within the oral tissues. The OOAC technology is assessed in this study toward better approaches in disease modeling and better therapeutics strategy for bone tissue engineering applied in dentistry.

## Plain language summary

Bone-related issues have been widely focused on in the field of dentistry due to oral cancers, trauma, injuries and the high incidence of periodontitis (a serious gum infection which causes bone damage and tooth loss). To overcome this condition, several strategies have been developed involving tissue engineering approaches and drug discovery. To provide better drugs for periodontitis, it is important to study the ways in which tissues and cells work together as well as the disease mechanisms, and cell interactions with drugs, other therapeutics agents, or biomaterials. For this, cell studies are needed, but the current research cannot replicate the disease environment and therefore cannot show exactly what happens in real sick areas. In this review, a new idea is explored called organ-on-a-chip technology, where scientists make small models that work like our organs, which could help them find better ways to treat dental and bone problems.

## Tweetable abstract

Monolayer cell cultures and animal studies cannot reflect real time microenvironment of the diseased sites. A microfluidic organ-on-a-chip technology relevant to dental and bone tissue engineering is a must to resolve the challenges for better therapeutics strategy.

## Graphical abstract

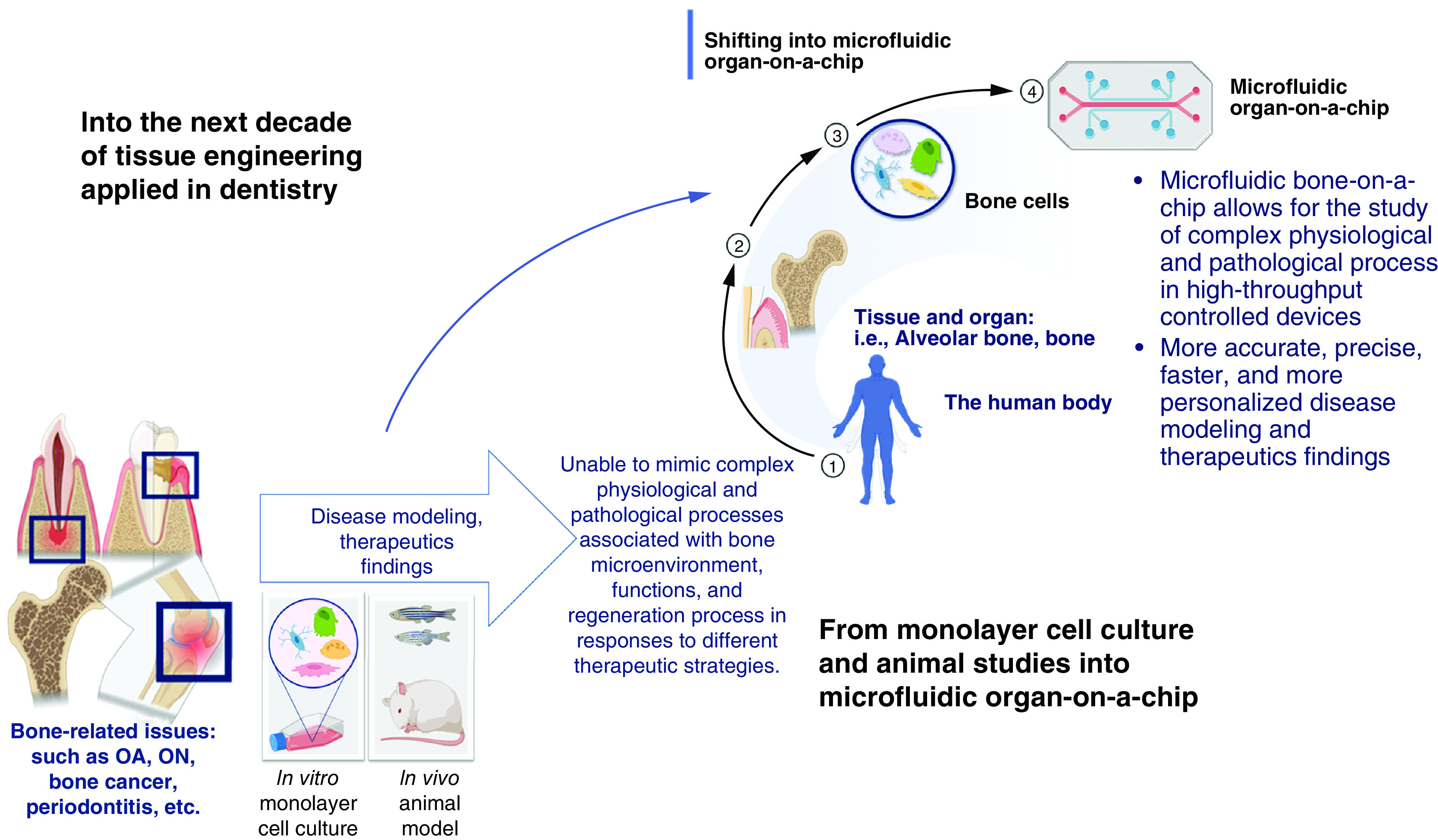

Bone is a dynamic tissue that is constantly capable of self-repair in order to maintain functionality throughout life through modeling during childhood and adolescence, balancing the formation-resorption process, and bone remodeling. Bone contains four types of cells, including osteoblast, osteoclast, bone lining cells and osteocyte. Bone cells are responsible for several vital functions such as supporting body and soft tissue structure, performing as the central region of hematopoiesis in adult humans, and being involved in mineral homeostasis [1–3]. However, this tissue may undergo damages caused by either congenital, age-related, or acquired in situations such as trauma, inflammation, infections, or surgical intervention that led to bone loss [4–7].

Nowadays, the most common bone-related issues are osteoporosis, osteosarcoma and bone metastases. As a systemic disease, osteoporosis has become a serious public health issue because of its high risk and prevalence, with approximately 200 million people affected worldwide [8–10]. Apart from osteoporosis, osteosarcoma is still the most common bone malignancy found. In these regards, bone is also reported as the third most frequent site of metastases after the lung and liver [11–13]. With respect to bone-related issue in dentistry, periodontitis is the second most common oral problem found in world populations after caries. Periodontitis is a multifactorial chronic inflammatory disease associated with biofilm dysbiosis. It is characterized by the progressive destruction of periodontal tissue, including the alveolar bone, leading to tooth loss. In view of the periodontal tissue, the alveolar bone is a part of the maxilla and mandible that forms and supports the tooth's socket [14]. It grows simultaneously with the process of tooth eruption and gradually disappears after tooth loss [3]. Numerous cases of periodontitis have been reported, in which according to the Global Burden of Disease Study (GBD) in 2019, periodontitis affected 1.1 billion people worldwide and the numbers still increase from time to time [15–18]. Bone also stated as one of the most frequently used tissues for transplantation. In the fields of orthopedic surgery, plastic surgery, maxillofacial surgery, and neurosurgery, more than a million people are treated for skeletal issues each year [19]. These bone-related issues are problematic for many scientists and clinicians and are, therefore, of interest to researchers.

Studying the physiological and pathological processes associated with bone and its response to different therapeutic strategies is a complicated task requiring knowledge of the cellular microenvironment that affects the behavior of cells in tissues or organs [20]. Especially when tissue engineering [21,22] is used as an approach to regenerate by creating functioning substitutes for damaged or defective tissues and organs, the underlying principle of cellular behavior in its microenvironment toward regeneration mechanism is important to increase the success rate of the therapy.

Bone tissue engineering (BTE) is a promising approach to enhance bone repair and regeneration via synergistic integration of biomaterials or scaffolds, cells and therapeutic factors [1,23,24]. In the context of maxillofacial applications, there is an extensive selection of sources and materials that can be utilized for the reconstruction of maxillofacial bones in the form of synthetic bone extracellular matrices which are generally known as scaffolds. These include autogenous, allogenic, xenogeneic, alloplastic, and engineered personalized grafts [25]. Within this context, the scaffold can provide interim mechanical integrity at the defect site until the bone tissue is repaired or regenerated and the process of tissue regeneration involved appropriate cell adhesion, proliferation and function [24–26]. Therefore, cell-based assay is the fundamental way to study and give clear information about this phenomenon.

To date back on the importance of cell-based assay, cell culture has become a necessary tool for discovering the fundamental mechanisms of cell assembly in tissues and organs, how these tissues function, and how that function becomes disrupted by an agent or a disease [27]. In correlation with the complexities, the approaches have been continuously developed and shifted gradually from two-dimensional (2D) monolayer cell culture to a three-dimensional (3D) cell culture system using a more realistic microenvironment called scaffolds. Nevertheless, both 2D and 3D cell cultures make certain sacrifices to facilitate experimental procedures and are still unable to reflect *in vivo* phenomena related to important organ features [27–29]. Although animal studies have been responsible for advancing knowledge in many biological studies, the models have various drawbacks, such as increasing experiment difficulty, reducing the feasibility of research, and failing to reproduce the complexity of humans [30]. Also, animal studies involve ethical issues and contradictive results from clinical trials, which is against the principle of basic biomedical research [31]. In today's society, there is a growing inclination toward the exploration of humanized *in vitro* alternatives as a means to replace animal research. Consequently, a pressing demand for the development of platforms that closely mimic human physiology and characteristics is increasing.

Recently, organ-on-a-chip (OOAC) based on microfluidic technologies has been proposed as an innovative cell-based assay tool in both basic physiological and regenerative research fields. Interest in OOAC has been intensified because OOAC combines chemical, biological and material science disciplines and offers more integrated aspects for a more complete understanding of tissue engineering and regenerative medicine. An OOAC approach has been chosen as one of the top ten emerging technologies by the World Economic Forum in 2016 [32]. The field of OOAC and micro-physiological systems has witnessed a substantial surge in interest, reflected by the publication of several commendable reviews in recent times [33–35]. Large-scale research at the national level has been conducted in some countries, and the application of this technology is expected in both practical and clinical use [36,37]. An OOAC is a micro-physiological system that recapitulates a human organ or tissue's physiology and functionality. This technology aims for effective and accurate medical, biological and pharmacological research, such as disease modeling and drug screening [38].

Microfluidic OOAC models have been developed over the last few years to recapitulate various organs and systems in the human body. As previously mentioned, OOAC has been studied for several organs, e.g., intestine [39], lung [40], blood vessel [41], liver [42], heart [43], kidney [44], bone marrow [45], brain [46], bone [47], and tooth [48] but published articles on bone tissue engineering and/or dentistry-related OOACs are still limited, though the subject is worth developing. This study aims to review OOAC to provide basic concepts, current applications of OOAC innovative technology in basic research, state-of-the-art, and future perspectives of OOAC in the field of bone tissue engineering, specifically the one relevant to regenerative dentistry.

## Methods

A literature search strategy using keyword database searches was applied, continued by the specified article's inclusion criteria. Two readers (MHS and IDA) then elaborated and summarized the findings. Articles from the PubMed, Science Direct, and Scopus databases were used in the study. Article investigations were conducted according to title, abstract, or full text that appeared using the keywords “lab-on-a-chip,” “organ-on-a-chip,” “microfluidics,” “microfluidic chip,” (“lab-on-a-chip” OR “organ-on-a-chip”), (“lab-on-a-chip” OR “organ-on-a-chip” AND “microfluidics”), and (“lab-on-a-chip” OR “organ-on-a-chip” AND microenvironment). All articles published in English before September 2022 that mentioned these OOAC keywords were included in this review. If the articles were found to be not experimental, review, or systematic review, the articles were then excluded from the study.

## Overview of organ-on-a-chip

The concept of “organ-on-a-chip (OOAC)” basically comes from an idea to resolve drug development problems that are happening these days. The increasing number of incurable diseases and the slowness or even failure of medicines to reach the clinic nowadays have become formidable obstacles for modern medicine. In fact, only 1 out of 9 drugs entering phase I will reach the market [49]. Drug development is usually divided into four main steps: discovery and advancement of potential compounds, *in vitro* and *in vivo* research, and clinical research; if the drug candidate shows safety and effectiveness in humans, the next step is to prepare a proposal for regulatory agency approval [50]. The entire drug development process is deemed inefficient resulting in unsustainable healthcare costs and medications with low efficacy and safety for the population [51,52]. The absence of efficacy and unanticipated adverse effects are the most frequent causes of drug withdrawal from the market [53]. Therefore, the entire process has been revised and the performance of *in vitro* tests in the preclinical stage including 2D and 3D cell cultures such as scaffolds [27] and organoids [54,55], as well as animal models are now highlighted and questioned [56,57]. As mentioned before, the criticisms of 2D and 3D cell culture focus on the inadequate physiological resemblance to healthy or diseased human tissue, lack of reproducibility, and limited to small-scale production, whereas animal models are time-consuming, expensive, and related to ethical issues. Furthermore, preclinical results are derived from non-human cells (cell culture and animal models), and their potentially misleading results are not replicated in clinical trials [58,59].

There is increasing demand to improve understanding of disease and accelerate the drug development process by finding more accurate models and alternatives to animal testing. In fact, according to the US Department of Agriculture, the US in 2018 utilized approximately 780,070 animals for *in vivo* testing. However, the outcomes of animal and human studies often fail to confirm each other [60,61]. Then, The Humane Research and Testing Act (HR 1744) and the US FDA Modernization Act of 2021 were approved by the US Senate in 2021, allowing drug manufacturers and sponsors to seek market approval based on the safety and effectiveness of alternative approaches to animal testing. At the same time, the European Parliament in the European region proceeded in the same manner with a resolution to support animal welfare and technological innovation [62,63]. Both included organ chips and micro-physiological systems as alternatives.

Organ-on-a-chip (OOAC) refers to a biomimetic micro-engineered system that mimics the structural and functional properties of humans at the organ level and even the organism level [64,65]. The basis of this emerging technology is a microfluidic chip that combines biology, materials science, and engineering to mimic the microenvironment of native tissue and organs *in vitro*. The platforms basically involved a microfluidic device, seeded with living cells, and maintained under constant fluid flow of biological fluids. The chip is also designed to work under stimulation and with other organ-relevant elements [37,66–72]. Microfluidics is the study and manipulation of microliter-scale fluids confined within micrometer-scale channels, chambers, or wells referred to as “chips” [73]. Microfluidic tools have attained a sophisticated level of development with the aim of comprehending *in vivo* conditions [74]. Combining technologies such as microfluidics and 3D cell cultures adds a new dimension to cell biology research, resulting in a more accurate simulation of the *in vivo* cell environment. It permits the examination of biological organs using minute volumes of fluid. They contribute to cell research by being easily miniaturized, user-friendly, sensitive, robust, and adaptable to a high throughput design [73,75,76].

The first primary objective of the earliest organ-on-a-chip models was to replicate vital physiological parameters, primarily in response to mechanical stimuli. Huh and co-workers published the first OOAC model developed using epithelial and endothelial cells to simulate the alveolar-capillary interface of the human lung. The device can replicate human breathing type and lung response to pathogen stimulation [77]. The OOAC is designated as one-chamber, multiarray, parallel, and serial organ chips [78]. Furthermore, by using various chip designs, cells can be organized into various natural tissue structures [78].

**Figure 1.**
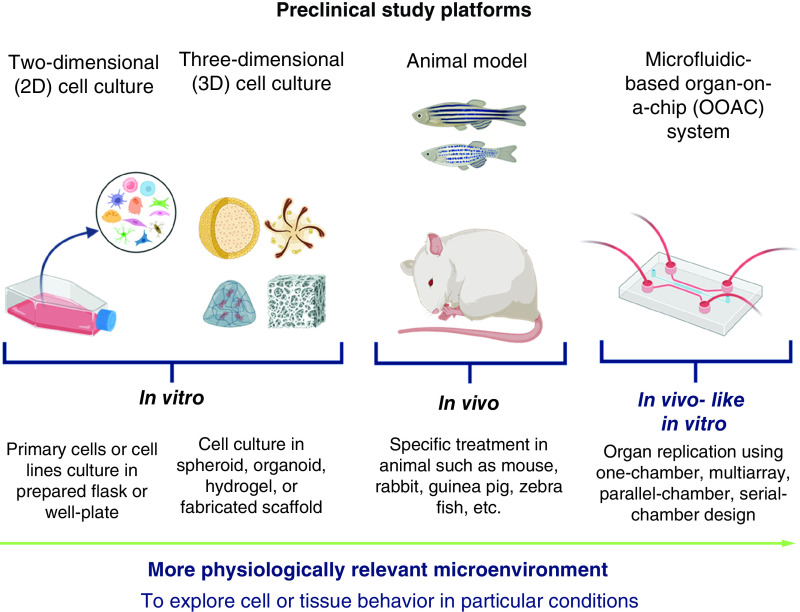
Preclinical study platforms.

Along with great interests and development, now OOAC as micro-physiological systems is built in different sizes and shapes [49], and it successfully established numerous models of healthy and diseased tissues and organs. The OOAC can be modeled to recreate a single organ-level structure and function, which is the most widely conducted in current research. The dimension of the OOAC approach was then enhanced by connecting two or more organ levels as a multi-organ chip, which came from an idea called “human/body-on-chip” that mimics whole-body physiology or pathology [64,78]. Multi-organ chips could be considered as the novel accurate model to study biodistribution, drug delivery systems, and metastases in cancer. This opened opportunities to develop several *in vivo*-like *in vitro* models for any desired organs or systems to study, as depicted in Figure 1 .

### The Potential of OOAC for Fundamental Research

The origin of OOAC comes from ideas combining microfluidics and tissue engineering. It was initiated with miniaturized total chemical analysis systems (μTAS), invented by Manz *et al.* in 1990. Further, with the advancement of knowledge and technology, the term “microfluidics” was applied [79,80]. In this context, microfluidics systems generate 10 to 100s of micrometer channels using a very small amount of fluid. Additionally, in tissue engineering, basic functional structures are formed by scaffolds, either alone or in combination with cells and/or signaling molecules, to replace or repair damaged tissue. The expected outcomes from merging these two technologies are to create a new and improved cell environment for cell culture mimicking *in vivo* physiological processes. The rapid growth of microfluidic-based cell culture technologies has been noticed in these two past decades, and these technologies are intended for bioscience and pharmaceutical research. With OOAC, it is possible to create environments that predict the *in vivo* trials, because when compared with the conventional two-dimension method, it accurately recapitulates the dynamic processes and 3D architecture of body tissues and organs [81,82].

**Figure 2.**
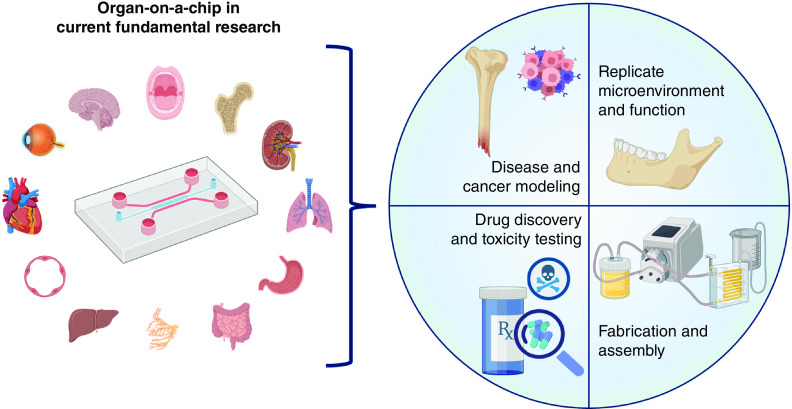
Current application of organ-on-a-chip in fundamental research, based on the literature search.

Although OOAC based on microfluidic technology has advantages such as being portable and cost-effective, reducing time and being better at mimicking tissue microenvironments, microfluidics technology needs more equipment, e.g., pumps, incubators, microscopes, and tools for a specific experiment [83]. So far, OOAC has mainly been used to mimic the physiological structures and functions of microenvironments and to model diseases and cancer, as well as for drug discovery and toxicity evaluation as illustrated in Figure 2.

#### Modeling physiological microenvironments & functions

The development of *in vivo*-like *in vitro* models such as OOAC integrates two distinct fields, microfluidics, and cell or tissue biology. By integrating the two, different human organ structures and functionalities can be built into a laboratory model that mimics the functions and responses of *in vivo* tissues and organs. Although OOAC technology cannot resemble a whole living tissue or organ, it is designed to organize a minimally functional unit of tissue or organ system that can better represent the aspect of human physiology [84]. Various human organs have been developed into OOAC platforms to recapitulate the functions of organs such as the intestine, lung, blood vessels, liver, heart, kidney, bone marrow, brain, bone, and tooth [39,48,85–107], as shown in Table 1.

**Table 1.** Developed organ-on-a-chip to mimic various human tissues or organs.

Study (year)	Chip name	Research aims	Cell type	Ref.
Zhang *et al.* (2021)	Epidermis-on-chip	To mimic normal histological features of the human epidermis	Normal human keratinocytes	[85]
Duc *et al.* (2021)	hNMJ on a micro structured microfluidic device	To create a mature, functional, and reliable human neuromuscular junction	Myoblast (muscle progenitor cells) and hiPSCs	[86]
Ahn *et al.* (2021)	MVEOC	To replicate the physiology of the endometrial environment	HUVECs, EECs and ESFs	[87]
França *et al.* (2020)	Tooth-on-a-chip	To replicate the architecture and dynamics of the dentin-pulp interface	SCAP	[48]
Zhao *et al.* (2020)	Biowire II chip	To create cylindrical cardiac microtissues for cell cultivation	Human pluripotent stem cell-derived cardiac tissues	[88]
Sontheimer-Phelps *et al.* (2020)	Human colon-on-a-chip	To replicate mucous bilayer formations and determine the accumulation of mucous	Primary patient-derived colonic epithelial cells	[89]
Bahmaee *et al.* (2020)	Bone microfluidic chip	To create an 3D environment and determine fluid shear stress of bone	hES-MPs	[90]
Mosavati *et al.* (2020)	Placenta on-a-chip	To reproduce a placental interface between maternal and fetal blood	Trophoblasts cells and human umbilical vein endothelial cells	[91]
Zhang *et al.* (2020)	3D Liver chip	To improve existing models used to mimic the liver	The liver cancer cell line (Hep-G2)	[92]
Shanti *et al.* (2020)	LN on-a-chip	To replicate the lymph node microenvironment	Human EB1, THP-1, and Jurkat cells	[93]
Rogal *et al.* (2020)	WAT on-a-chip	To mimic the structure of the human white adipose tissue-like structure	Human primary mature adipocytes	[94]
Jing *et al.* (2020)	Gut-vessel microsystem	To study the interaction between a host and a microorganism in the gut system	Human intestinal epithelial cells (Caco2) and HUVECs	[95]
Jalili-Firoozinezhad *et al.* (2019)	Microfluidic intestine-on-a-chip	To replicate human intestinal epithelium host–microbiome interactions	HIMECs and human intestinal epithelial cells (Caco2 BBE human colorectal carcinoma cell)	[39]
Petrosyan *et al.* (2019)	Glomerulus-on-a-chip	To recapitulate the functions and structure of the glomerulus	Human podocytes and human glomerular endothelial cells	[96]
Theobald *et al.* (2019)	Multi compartment microfluidic liver kidney organ on a chip	To recapitulate hepatic metabolism and renal bio-activation	HepG2 and RPTEC cells	[97]
Dai *et al.* (2019)	Disc-on-a-chip	To simulate and investigate disc metabolism and the *in vivo* disc microenvironment	Not explained but used a lumbar disc from a mouse	[98]
Albers *et al.* (2019)	Platelet aggregation on-a-chip	To quantify the aggregation of platelet patterns	HUVECs	[99]
Zhang *et al.* (2018)	3D human lung-on-a-chip	To recreate the human lung structure and functions and evaluate the toxicity of nanoparticles	Lung alveolar epithelial cells and human vascular endothelial cells	[100]
Wevers *et al.* (2018)	Human blood-brain barrier (BBB) on-a-chip	To replicate future therapeutic strategies	Human cell lines of brain endothelial cells, astrocytes, and pericytes	[101]
Jain *et al.* (2018)	Lung alveolus-on-a-chip	To recapitulate response *in vivo*, to recapitulate platelet-endothelial dynamics, and to analyze the inhibition of endothelial activation and thrombosis due to a PAR-1 agonist	HUVECs and primary human alveolar (type I and II combined) epithelial cells	[102]
Wang *et al.* (2017)	BBBoC	To mimic *in vivo* BBB characteristics in the brain	BMECs from hiPSCs and rat primary astrocyte	[103]
Banaeiyan *et al.* (2017)	VLSLL-on-a-chip device	To mimic the central vein of a liver lobule	Human hepatocellular carcinoma cells (HepG2) and hiPSC-derived hepatocytes	[104]
Musah *et al.* (2017)	Kidney glomerular-capillary-wall on a chip	To recapitulate the natural tissue or tissue interface of the glomerulus	hiPS cell-derived podocytes and primary human glomerular endothelial cells	[105]
Skardal *et al.* (2017)	Integrated three-tissue organ-on-a-chip (liver, heart, and lung)	To create a tissue organoid and tissue construct that integrates lung, liver, and heart in one chip	Human primary cells, including HSCs, iPSC CMs, vascular endothelial cells, lung epithelial cells, and fibroblasts	[106]
Lee *et al.* (2016)	Placenta on-a-chip	To reproduce the placental barrier	Human trophoblasts (JEG-3) and HUVECs	[107]

BBBoC: BBB-on-a-chip system; BMEC: Brain microvascular endothelial cells; EEC: Endometrial epithelial cells; ESF: Endometrial stromal fibroblasts; hNMJ: Human neuromuscular junction; HSC: Hepatic stellate cells; hES-MP: Human embryonic stem cell-derived mesenchymal progenitor cell; HIMEC: Human intestinal microvascular endothelial cell; hiPS: Human-induced pluripotent stem; HUVEC: Human umbilical vein endothelial cell; hiPSC: Human-induced pluripotent stem cell; iPSC CM: Induced pluripotent stem cell-derived cardiomyocytes; LM: Lymph node; MVEOC: Micro-engineered vascularized endometrium on a chip; SCAP: Stem cells from apical papilla; VLSLL: Very large-scale liver-lobule; WAT: White adipose tissue.

#### Drug discovery & toxicity evaluation

Toxicity is one of the main reasons for drugs failing in terms of either reaching the market or after it had already become available on the market. Therefore, conducting a preclinical toxicity evaluation of a new investigational drug (NID) is a very important step toward clinical application. Toxicity evaluation results from 2D cell culture and animal models sometimes cannot be determined during clinical tests due to unrepresentative preclinical trials or species differences [31]. To improve the precision of drug toxicity preclinical tests, the OOAC models have proven to be potentially novel approaches to studying drug toxicity in cells, tissues, and organs. The miniaturization and the dynamic process within the microfluidic chip considerably reduce needed samples and significantly improve the reliability and sensitivity of the tests [108,109]. Based on the literature search results, several OOACs have been developed for drug and toxicity evaluation [110–115], as shown on Table 2. It was confirmed from the investigations that OOAC is an excellent approach to studying drugs and toxicity evaluation. For example, in the study by Jang and co-workers [115], it was found that the toxicity test results were closer to *in vivo* experiments and proved to be an innovative tool for evaluating human renal toxicity. Jang *et al.* [115] measured the toxicity by the activity of cisplatin, a proximal tubule nephrotoxin, and P-glycoprotein ATP-binding cassette membrane transporter (Pgp).

**Table 2.** Search results on the use of organ-on-a-chips for drug development and toxicity evaluation.

Study (year)	Type of developed OOAC	Study overview	Ref.
Li *et al.* (2020)	A 3D human blood-brain barrier chip	The OOAC was used to study the neurotoxicity of INPM. It was shown that the platform effectively mimics the microenvironment and response of the human blood-brain barrier to INPM exposure. An INPM disrupts Keap1-Nrf2-ARE pathways in the blood–brain barrier.	[110]
Bovard *et al.* (2020)	Connected lung/liver-on-a-chip using cocultured normal human bronchial epithelial cells and HepaRG™ liver spheroids	It shows that acute and chronic toxicity of aerosol exposure from aflatoxin B1 (AFB1), as one of anti-tuberculosis agent, was reduced because of the presence of HepaRG™.	[111]
Kamei *et al.* (2017)	Integrated Heart/Cancer on a chip	The OOAC was used to study side effect of Doxorubicin as an anti-cancer drug on human healthy heart cells and liver cancer cells (HepG2) cocultured in a chip. The chip successfully demonstrated how Doxorubicinol, a toxic metabolite from HepG2 cells, is delivered and how it affects the heart cells	[112]
Nierode *et al.* (2016)	A microarray chip platform	The OOAC was used to compare the toxicity of 24 compounds in an undifferentiated and differentiated human neural progenitor cell line. The OOAC platform showed that the acute toxicity of five compounds, acetaminophen, 5-fluorouracil, retinoic acid, Doxorubicin, and pitavastatin, were different from two neural progenitor cell culture conditions.	[113]
Kwon *et al.* (2014)	Transfected enzyme and metabolism chip (Team Chip)	Team Chip was used to predict metabolism-induced drug toxicity or drug-candidate toxicity by manipulating the expression of human metabolizing-enzyme genes using THLE-2 cells and to reveal the specific enzymes related to the drug toxification process.	[114]
Jang *et al.* (2013)	Kidney proximal tubule-on-a-chip with human primary renal tubular cells	The OOAC was used to study nephrotoxicity. It was shown that the toxicity test results were closer to *in vivo* experiments and proved to be an innovative tool for evaluating human renal toxicity. It was measured by the activity of cisplatin, a proximal tubule nephrotoxin, and P-glycoprotein ATP-binding cassette membrane transporter (Pgp).	[115]

All investigations prove that OOAC is an excellent approach to studying drugs and toxicity evaluation.

INPM: Indoor nanoscale particulate matter; OOAC: Organ-on-a-chip.

#### Disease & cancer modeling

Cancer therapeutics require preferable and reliable experimental models [116]. One of the problems that lead to the slow development and invention of new anti-cancer therapies is the limitations of the preclinical models used to identify molecular, cellular, and biophysical changes as the critical features of human cancer progression [68]. Conventionally, researchers test potential anti-cancer agents in tumor cell culture, but the outcomes are insufficient without animal studies [117]. Animal studies involve tumor cells implanted subcutaneously in rodents. However, this model has widely accepted drawbacks because the model cannot mimic native-tissue cancer growth, responses to therapeutic agents, and the organ's microenvironment [118]. To resolve these challenges, researchers move to another model called *in vivo* orthotopic cancer models. These models are better at mimicking tumor growth and metastasis. Nevertheless, there are challenges involved in terms of identifying the role of the microenvironment in tumor growth and visualizing cell behavior over time, and the research is not conducted in humans [119,120]. Both 2D and 3D tumor models provide information about cancer cell interaction, migration, and invasion of the surrounding tissue microenvironment [121–124]. However, neither model can explain the role of mechanical forces related to fluid shear stress, hydrostatic pressure, and tissue deformation, which can affect tumor cells' behavior [125–129]. The latest *in vitro* models, called organoid culture technology, lack the capacity to represent the critical factors in cancer control and progression [130–132]. Furthermore, the difficulties involved in investigating metastases as a dynamic process of key cancer-related oncology issues have triggered significant interest in developing biomimetic *in vitro* models that can recapitulate cancer [133]. The development of cancer and disease modeling is also focused on the effect of therapeutic cancer strategies [68,134]. Thus, the OOAC approach is expected to fill the gap between preclinical studies and future clinical outcomes, and the models are expected to become the fundamental models used for studying cancer progression and metastases to obtain better future clinical studies and results.

Table 3 shows the search results on the use of OOAC for cancer modeling. It was noticed that scientists also use OOAC to study cancer growth, neovascularization, progression, migration, and metastasis [68,119,133,135–141]. The findings from the previous investigations suggested that the 3D microenvironment is crucial. As an example, a study by Montanez-Sauri [141] showed that the microfluidic chip 3D microenvironment significantly influences the development of the cells more when compared with 2D culture. Furthermore, all the previous research shows that the OOAC platform and approach can better model cancer in many aspects, depending on the research objectives. In fact, studies focusing on the application of organ-on-a-chip for bone cancer are limited because bone-on-a-chip systems are relatively new and have only been introduced recently in reviews, unlike other OOAC systems [142].

**Table 3.** Studies on cancer growth, neovascularization, progression, migration and metastasis using organ-on-a-chips.

Authors	Overview of the Study	Ref.
Chramiec *et al.* (2020)	To develop an integrated OOAC to reproduce bone Ewing Sarcoma and cardiac muscle to study the efficacy of anti-cancer drugs and cardiotoxicity and then compared the result from OOAC studies with the clinical trial results. The OOAC allowed the monitoring of cancer cell growth and assessment of anti-cancer efficacy and cardiotoxicity.	[135]
Liu *et al.* (2020)	To develop a micro-tumor using a microfluidic device to study anti-cancer drugs.	[136]
Weng *et al.* (2020)	To fabricate an integrate chip to analyse the effect of the potential toxicity of chemotherapeutics.	[137]
Oliver *et al.* (2020)	To prepare a microfluidic blood brain niche (μm-BBN) platform and study the tumor microenvironment and brain micro-metastasis.	[138]
Mamani *et al.* (2020); Xiao *et al.* (2019)	To use OOAC for cancer studies to recapitulate glioblastoma tumors and evaluate drugs for therapy.	[133,139]
Miller *et al.* (2018)	To develop a 3D human renal cell carcinoma-on-chip using primary human clear cell renal cell carcinoma and examine the ability of cells to stimulate tumor angiogenesis as a basis for pharmaceutical blockade studies.	[140]
Hassel *et al.* (2017)	To develop human orthotopic lung cancer-on-a-chip. The lung cancer-on-a-chip can be used to study lung cancer behaviours, rampant growth in a microenvironment, and tumor responses to therapy.	[119]
Montanez-Sauri *et al.* (2013)	To develop 3D microenvironment in a microfluidic chip and compare between 2D and 3D influences for the growth of human T47D cells. The microfluidic chip 3D microenvironment significantly influences the development of the cells more when compared with 2D culture.	[141]

OOAC: Organ-on-a-chip.

#### Fabrication & assembly

Microfluidics involves fluid behavior, precise control, and manipulation within small channel dimensions [72]. The OOAC system based on microfluidics consists of a microfluidic chip with chambers and channels where cells are cultured into an appropriate matrix or scaffold [20]. An OOAC based on microfluidics technology has some advantages, such as cost-effectiveness, easy accessibility and experiment flexibility. By using OOAC, experiments can be conducted by culturing or coculturing a small number of cells, with real-time on-chip analysis, using automation, and reducing reagent consumption and contamination [83,143]. Though it is cost-effective, the cost itself is a disadvantage of OOAC because of the need for specialized microengineering capabilities, cleanrooms, or pumps, which can be expensive [144]. Other disadvantages of OOAC are the design complexity, non-standard culture protocols, and complex operational procedure because it involves a small volume of reagent or liquid [83,145]. The way to conduct experiment using OOAC is sequentially from designing the chip, molding, seeding the cells, managing cellular growth, establishing functions, and calibration using imaging or several tests which include physical, chemical, and mechanical tests [144,145].

The OOAC system designs mentioned above share similar characteristics but depend on the objectives. The body of the chip houses all the channels, chambers, or other elements such as sensors, electrodes, or valves. The body part can use polymeric materials such as poly-dimethylsiloxane (PDMS), poly-methylmethacrylate (PMMA), polycarbonate (PC), polystyrene (PS), polyimide (PI), and polyvinyl chloride (PVC) and silicone [67,146]. A frequently used material in OOAC systems is PDMS, because it is cell friendly, inexpensive in a laboratory setting, biologically inert, gas-permeable, and has a non-toxic surface with low adhesion and qualities that support the systems [143,147–153]. However, PDMS has some drawbacks, so it opens opportunities to construct OOAC systems from the other potential materials mentioned above. Most microfluidic device fabrication uses different techniques, such as etching, nanofabrication, replica modeling, injection molding, lithography microcontact printing, and the emerging method of using 3D printing [83,147,148,154–161].

Several materials, such as natural or synthetic polymer substances, are used as membranes or scaffolds. These membrane and scaffold manufacturing techniques include electrospinning [162], 3D printing [163], stereolithography [164], fused deposition modeling (FDM) [165,166], selective laser sintering (SLS) [167,168], bio plotting [169], salt leaching [170,171], and freeze drying [172,173]. Other potential materials for OOAC can also be utilized, such as silkworm (Bombyx mori), agarose hydrogel, Teflon, acrylonitrile butadiene styrene (ABS), polyurethane methacrylate (PUMA), polyethylene glycol (PEG), polyhydroxyalkanoates (PHA), gelatin methacrylate (gel-MA), poly(polyol sebacate) (PPS), and styrene ethylene butylene styrene (SEBS) [83].

Although there have been several fabrication methods available for the development of OOAC, there are still engineering limitations to reaching the full complexity of human physiology. For example, numbers and sizes of vessels, tubes, and ducts in human tissues and organs are still too complex to be fully recreated in engineered systems. Even the development of relatively simple channel networks can be challenging to operate vigorously and efficiently. Different fabrication materials and methods will result different quantity or amount of raw material processed within a given time, which is required to cover variabilities that arise from biological heterogeneity.

**Figure 3.**
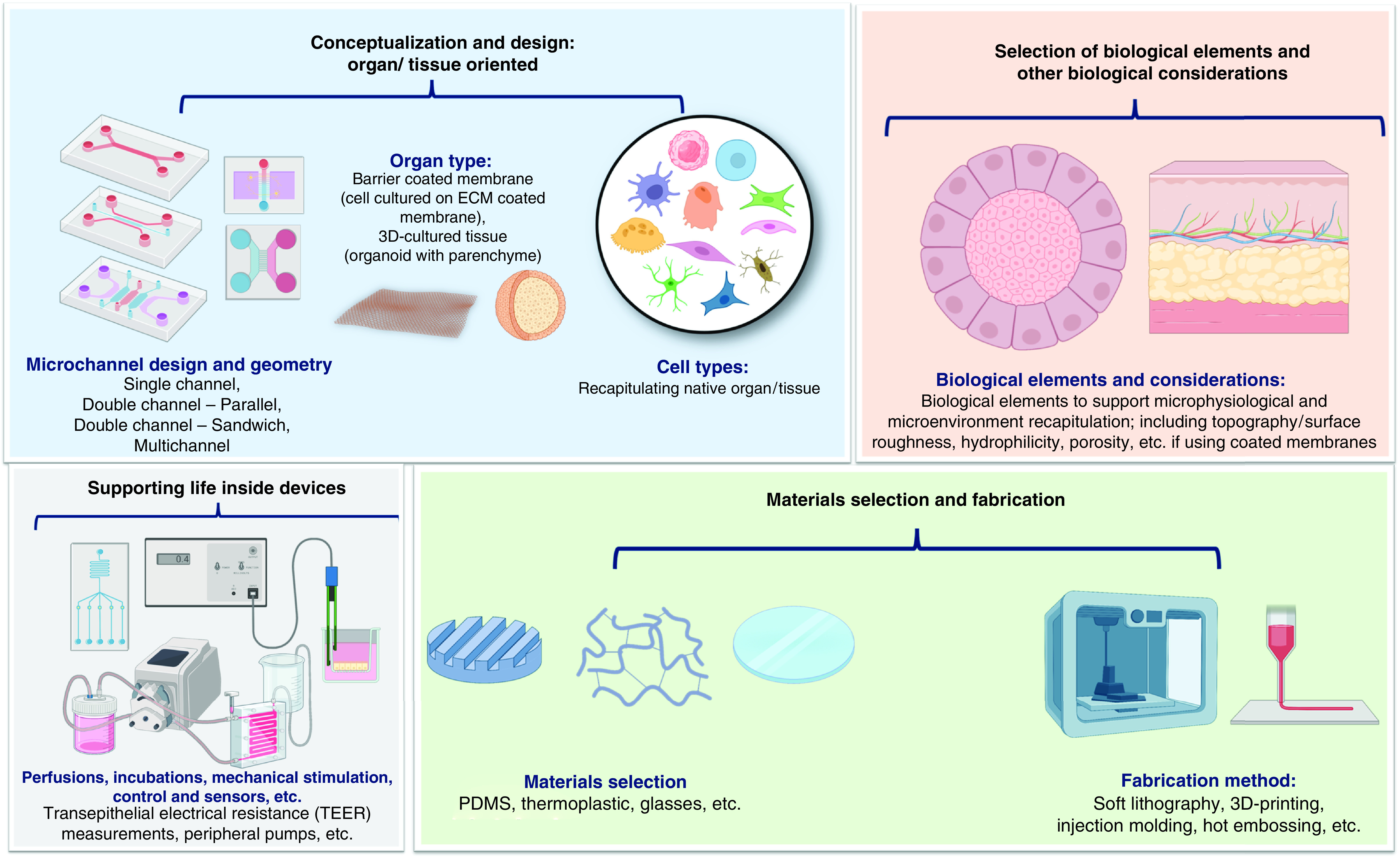
General considerations to design, fabricate, and assembly microfluidics based organ-on-a-chips. ECM: Extracellular matrix; PDMS: Poly-dimethylsiloxane.

The OOAC based on microfluidics uses flow mechanisms and various types of cells derived from humans and animals, e.g., mice used as single or multiple cells within the system. Flow mechanisms are differentiated into two types, active and passive [155]. The active flow mechanism uses a syringe and peristaltic pumps, whereas the passive flow mechanism depends on gravity-driven flow [174–180]. The type of cells used is based on the organ that is targeted for replication. Nowadays, developments have led to the use of stem cells, including multipotent mesenchymal stem cells (MSCs), pluripotent embryonic stem cells (ESCs), and induced pluripotent stem cells (iPSCs) [180–182]. Stem cells have potential because they can differentiate into cellular subtypes [183,184]. Finally, since the behavior of cells changes depending on triggers within the human body, to achieve complete functionality, stimuli is given to the microfluidic chip. Researchers involve a specific condition for organs/tissues, including chemical and mechanical stimuli to observe the responses of living cells, e.g., pressure, flow rate, pH, osmotic pressure, toxins presence, nutrient content, drugs including chemotherapy, and radiation [83,185,186]. Figure 3 provides an overview on the generic considerations to design, assembly, and fabricate microfluidics based OOAC.

### State-of-the-art in OOAC for dentistry & bone tissue engineering

Humans have more than 200 bones, and these organs may undergo damage or losses caused by accidents, extreme sports, aging, and/or bone-related conditions and disorders [187]. On the other hand, bone also has excellent capacity to regenerate and spontaneously repair damage [188,189]. Despite its excellent regenerative capacity, when there is a large critical defect in bone, its self-repair capability needs to be enhanced. In this point, TE rises as a well-proven technique in regenerative medicine [187] to help bone to regenerate using scaffold as synthetic ECM, signaling molecules, and cells, either alone or in combination. As the branch of TE, BTE specifically focuses on bone regeneration by combining multiple aspects of biology, engineering, material science, clinical medicine, and genetics to construct biological substitutes, i.e., scaffold, to promote bone regeneration [190]. The scaffold must mechanically, biologically, and physically mimic the dynamics and functionality of the extracellular matrix (ECM) of a specific tissue [191]. However, bioengineered scaffold is still massively developed under static cell culture condition, with its restriction in cell to cell and cell to ECM interactions.

The static condition affected cellular morphology as a consequence of insufficient physiological environment replication [191,192]. This is contradictory with the situation wherein ECM dynamics should play important roles in regulating tissue-specific cellular responses, thus affecting regeneration process, tissue formation, wound healing, and disease progression. In such a way, it is inadequate to depend solely on static conventional cell culture for accurate assessment of drug disposition, efficacy, and toxicity within the human body [64,193,194]. Therefore, fundamental research on cellular behavior should be conducted within better platforms that can mimic the dynamics of the bone as the primary interest tissue. Along with that, a microfluidic OOAC is expected to resolve the challenges. By integrating the principles of microfluidics, tissue engineering, and lab-on-a-chip (LOC) technologies, microfluidic-based OOAC incorporates miniaturized cell-culturing microenvironments with microchannels and compartments that replicate the natural environment of human cells [195].

#### Dentistry-related OOAC

**Figure 4.**
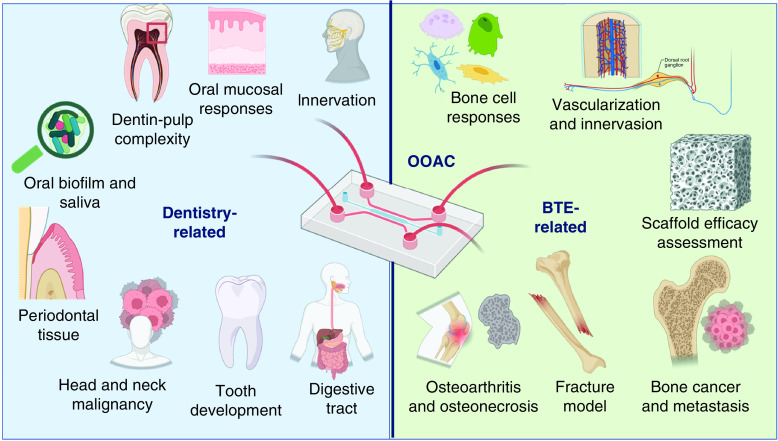
Recent developments and applications of dentistry- and bone tissue engineering related organ-on-a-chips based on the search results in this study. To this date, the OOAC was used to study biofilm and saliva [187–211], dentin and pulp complex [212–216], oral mucosa [213–220], periodontal tissue [221–224,233], and oral malignancies [225–229]. Some other groups developed OOAC to study digestion mechanism [230], innervation [231], tooth germs and oral cell differentiation [212,232]. It is also noticed various applications of OOACs in BTE not only to study diseases in the cell levels, but also in real time tissues environments [234–268]. BTE: Bone tissue engineering; OOAC: Organ-on-a-chip.

Regardless of the progression of severe and high periodontitis prevalence, there are still few published works on OOAC in relation to the TE model for dentistry, particularly for alveolar bone tissue engineering. Figure 4 summarizes the search results from this study regarding OOAC for dentistry that have been developed and investigated by several research groups. With respect to dentistry, it was found that OOAC has been used to study biofilm and saliva [195–211], dentin and pulp complex [212–216], oral mucosa [213–220], periodontal tissue [221–224], and oral malignancies [225–229]. Some other groups developed OOAC to study digestion mechanism [230], innervation [231], tooth germs and oral cell differentiation [232].

The oral cavity is home to a highly varied microbial community [196]. Oral microorganisms can colonize both on biotic and abiotic surfaces [197,198]. The colonization and growth are initiated by the adsorption of salivary pellicle proteins, which are present in saliva, on all available oral surfaces [199,200]. Following that, then accumulates, and forms structures called biofilms. Oral biofilms are the primary cause of a wide range of oral conditions, including dental caries, periodontal disease, implant-related infections, and candidiasis [201,202]. Oral biofilms are strongly related to saliva because saliva plays significant roles in maintaining oral soft and hard tissue health such as cleansing activity and remineralization [202,203].

To study complex mechanism of oral biofilm and saliva, different research groups developed OOAC. For example, Rath *et al.* developed a flow chamber model for dental implant materials assessment. The study proved that biofilm from bacteria such as *Streptococcus gordonii*, *Streptococcus oralis*, *Streptococcus salivarius*, *Porphyromonas gingivalis*, and *Aggregatibacter actinomycetemcomitans* can be formed on the surface of titanium implant placed within the model. Rath *et al.* concluded that flow chamber model is a promising approach to replicate biofilm formation and antibacterial effect of dental materials [204]. Kristensen *et al.* designed a 3D printed resin flow-cell for *in situ*-grown biofilm analysis under shear-controlled flow. The study observed the impact of stimulated salivary flow (5 mm/min) to pH changes in biofilm. The model proved the importance of flow on pH changes and targeted to be an *in vitro* model to measure pH of biofilm [205]. Two studies by Kolderman *et al.* [206] and Luo *et al.* [207] involved microfluidics technology to quantify the structure of oral biofilms after being exposed with a reagent for biofilm interventions. For this purpose, Luo *et al.* [207] combined a novel in-house developed image analysis program called Biofilm Architecture Inference Tool (BAIT). In another study, Gasthi *et al.* used one-chamber microfluidic platform to investigate the chemical and hydrodynamic influences on biofilm pH variations [208]. To study the dynamic interaction between bacterial species, Jalali *et al.* [209] utilized a microfluidic-based co-culture system combined with time-lapse imaging to investigate biofilm dynamic interactions. Another model by Lam *et al.* [210] has also been developed to observe the effect of microenvironmental factors on long-term dental bacteria growth and biofilm development using high-throughput microfluidic devices which allows quantitative analysis. Furthermore, Thita *et al.* introduced a systematic and automated design of a microfluidic compact disc (CD) to investigate the electrochemical property changes of saliva after mixing with various types of mouthwashes using electrical impedance analysis. The developed model has demonstrated the potential of salivary theragnostic research [211].

Microfluidic based OOAC also developed to study dentin-pulp complexity after exposure to materials. Franca *et al.* developed a tooth-on-a-chip which replicated the dental pulp interface. The study involved clinically standard materials used in dentistry, and the model was found suitable as a novel platform to study dental cells after material exposure [212]. Another study by Hu *et al.* involved dentin disc within tooth-on-a-chip to evaluate the influence of the dentin barrier and permeated silver diamine fluoride on cell viability [213]. Rodrigues *et al.* developed tooth-on-a-chip to mimic the biomaterial-biofilm-dentin-pulp interface. They observed the interaction of bioactive dental materials with the dentin-pulp complex on a model of restored tooth and real time assessment to antimicrobial effect of calcium silicate cements as material for vital pulp therapy at the interface [214].

In addition, for the case of dentin hypersensitivity, as a part of the pulp, odontoblast plays a crucial role in it. Anyhow, none of the *in vitro* models has ever created to mimic the growth of odontoblast in dentinal tubules. In response to this, Niu *et al.* then developed a parallel microfluidic platform consisting of various sized microchannels. They aimed to determine the optimal size to induce odontoblast processes [215]. Another model was also developed by Qi *et al.* to study angiogenesis sprout for pulp regeneration purpose. They used microfluidic system with tapered microchannels seeded with endothelial and stem cells to explore optimal conditions to enhance angiogenesis [216]. In another experiment, Zhang *et al.* utilized angiogenesis microfluidic chip to study the significance of Sema4D–plexin-B1 signaling in the recruitment of dental-derived stem cells during angiogenic sprouting and the formation of blood vessels [217].

Soft tissue responses to materials used in dentistry are critical point for the development of a novel dental material. Standards for biocompatibility and cytotoxicity have been developed but the conventional cell cultures are not capable in mimicking multi-layered cell configuration [218]. Accordingly, Ly *et al.* developed oral mucosa-on-a-chip as an approach to resolve this problem. They evaluated the oral mucosal reaction to various 2-hydroxylethyl methacrylate (HEMA) concentrations and compared the platform with conventional cell culture [218]. With the same mucosal platform, Rahimi and co-workers studied the effect of dental monomer HEMA and *Streptococcus mutans* exposure to mucosal construct [219]. Regarding dental material exposure to oral mucosa, Koning *et al.* developed a multi-organ-on-chip which connects gingiva and skin, to examine metal exposures to oral mucosa. They observed from the chip that metal exposure can result skin inflammation from activation of the immune system [220].

Inside an oral cavity, periodontium gains specific attention to both oral health clinicians and researchers. A healthy periodontium provides good support to help maintain the tooth's position and normal function. The periodontium is composed of four principal components, i.e., gingiva, cementum, periodontal ligament (PDL), and alveolar bone. These components are different in some respects, such as location, biological composition, chemical composition, and tissue architecture, but all these components are integrated [233]. The integrity of these components represents the key success to all conservative, endodontic, and prosthetic therapies and becomes initial requirement for clinical success evaluation [221].

Several periodontium related OOACs have been developed to study periodontal tissues. A group of Vurat *et al.* developed a 3D-bioprinted microtissue model to mimic the interface between periodontal ligament and alveolar bone. The developed model was used to assess drug uptake and toxicity and proved to be potential as an *in vitro* platform to study PDL [222]. Meanwhile, regarding maintenance of periodontal homeostasis and prevention for subepithelial tissue against harmful agents, gingival epithelium-capillary interface is crucial. For this, Jin *et al.* developed a microfluidic epithelium-capillary barrier that closely mimics gingival epithelial barrier. The model was constituted to be suitable for periodontal soft tissue and drug delivery study [223]. Makkar *et al.* also developed microfluidic platform called gingival crevice-on-chip and aimed to simulate the gingival crevicular features, both in healthy and diseased condition. The model was observed to be a potential device to assess complex interaction within periodontal diseases [224].

Malignancies such as head and neck cancers can arise from cells within the mucosal surface of oral cavity [225]. Head and neck cancer has become problematic for our population. This type of cancer ranked sixth among the most common solid tumors worldwide, with head and neck squamous cell carcinomas (HNSCC) as the most common type [226,227]. The HNSCC has poor treatment outcome, and the overall survival was low. To get better understanding of HNSCC as a tissue derived cancer, Bower *et al.* developed a miniaturized tumor culture system. They detected that microfluidic system can maintain HNSCC for 48 hours [228]. Furthermore, Jin *et al.* developed a microfluidic-based perivascular tumor model to assess tumor drug sensitivity and in parallel investigate the toxicity within the endothelium. They found that the model had potential for personalized tumor medicine application in clinical settings [229].

In addition, some models have also been developed to study digestion process, innervation, and oral cells differentiation. De Haan *et al.*, for example, developed miniaturized enzymatic digestive system to replicate digestive functions within three-compartment enzymatic digestion consist of mouth, stomach, and small intestine. They applied some compounds and monitored the enzyme kinetics from the first reaction inside the microfluidic system. They discovered positive results on the enzyme kinetics monitoring system inside the developed microfluidic device [230]. Regarding tooth development, Pagella *et al.* has conducted an experiment to appraise the utility of a microfluidics device for co-culturing mouse trigeminal ganglia and tooth germs at various developmental phases. The study proved that microfluidics system is a useful instrument to investigate how neurons behave as orofacial tissues and organs were developed [231]. In another study, Kang *et al.* developed a microfluidic device system to explore oral epithelial-mesenchymal interactions as a key role in human tooth development [232].

#### Bone tissue engineering related OOAC

The field of BTE enables us to resolve the structural issue by combining two crucial components: osteoprogenitor cell culture and scaffolding materials. This combination serves as a template for cell proliferation, production of bone-like extracellular matrix, and specific required chemical cues for bone development [234–236]. Some microfluidic organ-on-a-chip technologies have been created to understand the biology of bones as well as bone-related diseases and treatments [142].

Related to bone cell functions, Babaliari *et al.* developed a flow-controlled system to determine the bone cells responses, such as orientation, proliferation, and osteogenic differentiation, after the application of various flow rates. The system was found to be beneficial for the tunable control of the cell microenvironment, which guided cellular activity involved in bone repair [237]. Meanwhile, Sheyn *et al.* also developed bone-on-a-chip system with constant flow in comparison with static culture. The study involved an optical imaging technique for cell survival, osteogenic differentiation, gene expression analysis, and immunostaining for osteogenic markers [46]. Another study by Middleton *et al.* has successfully cultured osteocytes and osteoclast precursors within a microfluidic co-culture system. By the construct, they aimed to examine osteoclast precursor responses to mechanically stimulated or unstimulated signals produced by osteocytes, as well as osteoclast modulation by osteocyte mechanical sensitivity. This platform helps mechanical transduction studies be more relevant [238].

By involving hydrogel technology, Nasello *et al.* developed a system to mimic osteoblast development into osteocytes using primary human osteoblast seeded in type I collagen hydrogel with modified cell densities. Nasello and teammates observed that cell densities applied within bone-on-a-chip affect the proliferation, alkaline phosphatase (ALP) activity, and production of osteocyte or osteoblast specific marker [239]. With the same approach as Nasello *et al.*, Bahmee *et al.* developed osteogenesis-on-a-chip with physiologically relevant flow conditions which incorporates 3D polymer scaffold. The flow on this approach provided human embryonic stem cell-derived mesenchymal progenitor cells (hES-MPs) to proliferate, differentiate, and produce extracellular matrix [240].

In relation to BTE, different approaches can be made to study bone vascularization and innervation. Jeon *et al.* developed a human 3D microfluidic model to investigate organ-specific human breast cancer cell extravasation into bone and muscle microenvironments. The bone microvasculature was reproduced using a tri-culture of human bone marrow mesenchymal stem cells (hBM-MSCs), osteogenically differentiated (OD) hBM-MSCs, and human umbilical vein endothelial cells (HUVECs) embedded in fibrin gel. The results showed functional microvascular network was developed along with vasculature specific markers such as vascular endothelial (VE) cadherin and zonula occludens (ZO)-1. Additionally, mature bone tissue formation was confirmed along with secretion of bone protein such as osteocalcin (OCN) and bone ALP [218]. In this regard, bone is well-innervated by peripheral nerves, which cooperate with the central nervous system. The factors released by nerve fibers have been found to be directly linked to bone cell functions [241,242]. Moreover, to study the role of innervation in skeletal development, Silva *et al.* developed a microfluidic device to examine the impact of dorsal root ganglion (DRG) neurons on the capacity of MSCs to differentiate into osteoblasts. Using a bone-like microenvironment approach, direct interaction between DRG neurons and MSCs increased the osteogenic differentiation of MSCs into osteoblast via regulating the production of Cx43 and N-cadherin and activating the canonical/-catenin Wnt signaling pathway [243].

Microfluidic-based systems have also been utilized to accelerate bone regenerative materials development as well as develop miniaturized bioreactors with high accuracy [119,244,245]. Lee *et al.* [132] prepared a microfluidic 3D bone tissue model for testing the performance of designated biomaterials fabricated by inkjet-printed micropatterned containing antibiotic and biphasic calcium phosphate (BCP) nanoparticles as a filler, dispersed in a polymer matrix to accelerate wound healing and prevent bacterial infection. The experiment showed the biomaterials can kill bacteria and at the same time enhance osteoblast production. The model developed has the potential to reduce the number of samples and culture experiments, while providing *in situ* monitoring for biomaterials-bacteria interactions [246].

In the context of bone regeneration, cell migration is a crucial phase in numerous regenerative processes [247]. For this, Movilla *et al.* has assembled a bone fracture model intended to analyze the impact of ECM properties and growth factor gradients, as well as quantitatively examine the migration characteristics of human osteoblasts (HOB) on collagen-based matrices. The platform was revealed as a promising tool to mimic bone healing microenvironment. The platform was also capable for an *in vitro* assessment and quantification of various biophysical and chemical parameters that affect osteoblastic cells migration [248].

This study also resulted in a considerable number of studies concentrate on cancer and its metastasis as a complex and multistage process [225]. In fact, bone metastases occurrence still rises and became the third most common location for cancer metastases after the lung and liver [249–252]. Bone cancer metastases can significantly decrease patients' quality of life due to skeletal-related complications [253]. Various models of OOAC grown into effective instruments for modeling cancer metastasis and understanding unique interactions between cancer cells and vital regulators of cancer niche [254]. Therefore, a set of studies using microfluidic OOAC have been focused on cancer metastases to bone. Conceição *et al.* established a metastasis-on-a-chip that replicates neuro-breast cancer interaction in a bone metastatic context, permitting both selective and dynamic multicellular paracrine communication between sympathetic neurons, bone tropic breast cancer cells, and osteoclasts. Experimental results showed synergistic paracrine signaling between sympathetic neurons and osteoclasts induced pro-inflammatory cytokines, which indicated increased aggressiveness of breast cancer [254]. Meanwhile, Mei *et al.* developed the first bone metastasis microfluidic tissue model consisting of a simulated blood vascular environment in which cancer cells can extravasate and a bone environment model that can deliver mechanical forces to cells. The study aimed to explore the function of osteocytes in the mechanical regulation of breast cancer bone metastases. The device allowed integrated stimulatory bone fluid flow and proved that mechanical stimulation of osteocytes reduced extravasation of breast cancer [255]. Both chips developed by the group of Conceição and Mei can be used to observe some processes at the bone metastatic microenvironment.

Nowadays, apart from bone cancer, osteoarthritis (OA) and osteonecrosis are also problematic. It was reported that OA is a degenerative cartilage disease and a major contributor to disability that affects millions of people worldwide [256,257]. In recent years, there has been some fascinating progress in understanding the basis of OA, as accumulating data reveals that OA is a whole-joint disease affecting all joint components, i.e., cartilage, synovium, subchondral bone, and related muscles [258–261]. In view of this, a model that accurately captures the whole-joint disease aspect of OA in humans is required. Makarczyk *et al.* developed an OOAC called “miniJoint”, consisting of an osteochondral unit (OC), adipose tissue, and inflammation-inducted synovial fibroblast-like tissue (SFT), to investigate its potential to develop novel OA therapeutics intervention. Therapeutics intervention has been proved to be effective in reducing inflammation and showed an increased production of glycosaminoglycan. The model by Makarczyk *et al.* was concluded to be potential and can be used to develop novel OA drugs [262].

Osteonecrosis, which predominantly affects young adults (under 50 years of age), is a progressive condition characterized by cell death, fracture, and collapse of the affected area due to inadequate blood supply. The prevalence of osteoarthritis, osteonecrosis, and the necessity of total hip arthroplasty (THA) have been rapidly increasing [263,264]. Some drugs can induce this condition, such as corticosteroids, the second most common cause of osteonecrosis of the femoral head (ONFH) [265,266], and specifically related to dentistry is bisphosphonates in medication-related to osteonecrosis of the jaw (MRONJ) [267]. An OOAC technology has been now applicable to assess osteonecrosis. In the study by Li *et al.*, a microfluidic OOAC was assembled to investigate the effects of various therapies on bone microvascular endothelial cells (BMECs) and the pathophysiology of steroid-induced osteonecrosis. The microfluidic system successfully proved glucocorticoids damage BMECs through the production of cleaved caspase 3/7 [268].

Based on this study, it can be acknowledged that numerous review articles on the developments of generic organ-on-a-chips have been published, as well as some platforms directed for specific to various organ systems. However, for bone tissue engineering the developments of OOACs, either the ones purposively designed and fabricated for general BTE, or the ones specifically directed for dentistry, deserves more attentions because of the high complexity of the bone tissue and because this field is worth developing. When the high prevalence of periodontitis with its progressiveness is also taken into considerations, the shifting approach to resolve challenges for bone-related diseases in dentistry using alveolar-bone-on-a-chip has been in the hands of bone tissue engineers, researchers, and dental clinicians.

## Conclusion

The OOAC systems related to BTE in dentistry are worth developing. The OOAC approaches are expected to fill the gap between preclinical studies and future clinical outcomes. Microfluidic OOAC models have been developed over the last decade to mimic tissues, organs, and systems in the human body to solve problems related to monolayer cell cultures and animal laboratory methods. Although research and development on OOAC in dentistry- and bone-specific are still limited, soon, OOAC approaches is predicted to be extensively used and direct trends in dentistry. It is because OOAC can better recapitulate physiological structures and functions, model disease and cancer, and provide more accurate data to support drug discoveries and other therapeutics strategies.

## Future perspective

**Figure 5.**
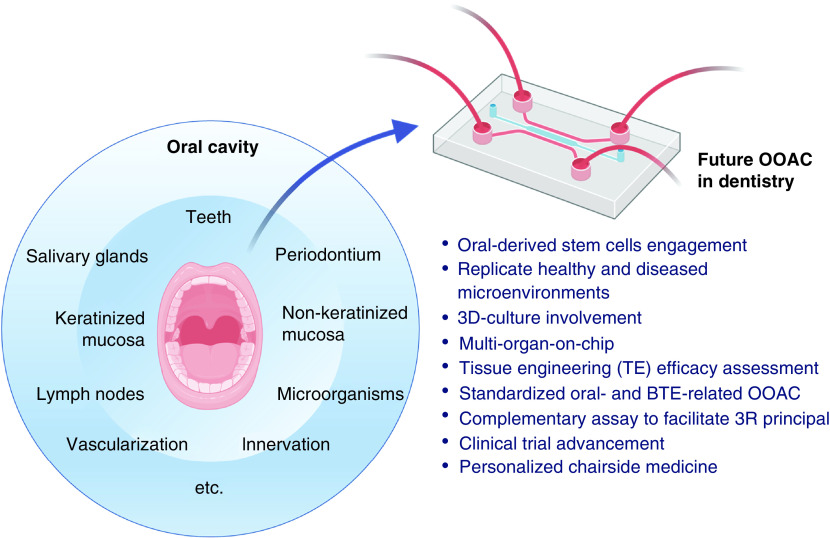
Future directions on the use of organ-on-a-chip in dentistry. BTE: Bone tissue engineering; OOAC: Organ-on-a-chip.

Organ-on-a-chip is categorized as a cutting-edge research tool in biomedical areas, especially in dentistry. Recent OOAC developments have been proven to be successful in mimicking real time physiological and pathological microenvironments. Designing *in vivo*-like *in vitro* models as shown in OOAC for both healthy and diseased conditions is a strategic option to assess and accelerate novel therapeutics discoveries. Since OOACs can also be designed to recapitulate either a single- or multi-organ system in only one small integrated device, the development of OOACs will significantly impact research, development, and valorization process in the field of biomedicine, or to be more specific, in tissue engineering and its applications in dentistry. Recent advances in OOAC have also shown that it may be possible to imitate wound healing or remodeling process after graft or plate implantations and augmentations, dental implant placement, as well as other bone-related surgeries in dentistry. In the next decade, the use of OOAC in dentistry is expected to provide more accurate, precise, faster, and more personalized solutions for unpredictable diseases or infections, as shown in Figure 5.

Nowadays, by applying microfluidic OOAC approach, the possibility to develop various organs in oral cavity is widely open. By OOAC, organs and tissues in oral cavity such as tooth, oral mucosa, temporomandibular joint, maxilla and mandibula, as well as periodontium which includes cementum, PDL, gingiva, and alveolar bone can be actualized for regenerative dentistry. The possibility for this has been on lab bench following the use of stem cells to control cell differentiation into desired cell types which have been proven. In addition to that, because the use of primary cells from a specific human organ to recapitulate desired organs is relatively difficult to retrieve, it has become an open area for us to shift into the use of oral-derived MSCs for OOAC studies, due to their easiness to isolate and manage, without altering their native behavior *in vitro*. In view of this, extensive research in combining OOACs technology with stem cell technology should be accelerated with respect to oral MSCs. As it has been reported previously [269–273], differentiation capacity of oral MSCs covers the ability to differentiate into nerve cells, odontoblasts, cementoblasts, myoblasts, hepatocytes, adipose tissue, melanocytes, osteoblasts, chondrocytes, and endothelial cells. For tissue regeneration, these stem cells have the potential to regenerate some organs such as brain tissue, eyes, liver, heart, spine, bone, cartilage, skin, muscle, and teeth [271–274]. This breakthrough is useful to recapitulate organs in the oral cavity, as well as bone as the key factor in bone tissue engineering.

Application of 3D cultures such as hydrogels, organoids, spheroid, and 3D bioprinted object into OOAC devices is essential to better mimicking ECM and directing cell behavior and communication [76,250]. Thus, it is approximated that the use of OOAC in dentistry will increase significantly to overcome disease complexity in the oral cavity. Moreover, OOAC technology will be growing toward multi-organ chips. A multi-organ chip is an integrated microfluidic chip with more than one organ structure and functions. These synchronous chips can be adjusted to observe the possibility of oral mucosal vaccines, drugs, or biomaterials side effects, study cancer metastasis, and understand the pathophysiology of systemic diseases with oral manifestations. A broader idea of multi-organ-on-chip may also lead to human-on-a-chip, replicating integration of all tissues and organs in the human body.

The future development of OOAC technology will also focus on the fabrication and assembly methods. It is anticipated that soon, the advancement of OOACs may lead to standardized microfluidic chips and protocols for their laboratory applications, which require standardized materials, flows, chip size and types, tools, reagents, sensors for monitoring, and methods of analysis. These standardized protocols are expected to ensure better research reliability and reproducibility. Consequently, to achieve the objectives of OOAC technology, inter and transdisciplinary approaches are needed by integrating various fields of study, such as biomedicine, bioengineering and biotechnology, dentistry, engineering, medical sciences, molecular biology, material sciences, and data analysis.

Standardized protocols are also relevant to challenges in OOAC commercialization. So far commercial use of OOAC systems has been focused on drug development, to estimate both efficacy and toxicity for humans in preclinical trials. The commercial use of OOAC has been a huge advantage in allowing a company to choose therapeutics candidates that have a higher chance of becoming approved drugs, thus it has shifted and revolutionized preclinical stages [275]. A lot of laboratories have also initiated start-ups for OOAC commercialization. However, how to create OOAC to become compatible with various imaging system, analytical instruments, robotics, and mass production, as well as to make OOAC user-friendly so that it can be widely adopted by non-specialist end users have been challenges for OOAC commercialization.

Finally in the future, organ-on-a-chip technology carries expectations that could revolutionize preclinical, clinical, and market stages of drugs and medical devices development, in TE and dentistry. In preclinical stage, OOACs can be a complementary technology to previous tools which provides more ethical options to facilitate 3R principle (reduction, refinement, and replacement) in animal studies with statistically insignificant results [276–279]. In the clinical stage, the most risky and expensive process, OOACs with continuous research and development will adapt as a supportive assessment for clinical trials before it can totally change or replace the current clinical trial phases. Further, using patient-specific cells allows identification of significant variances related to genetic diversity, race, gender, and age, rather than treating future patients as a homogeneous group. This unique approach also opens the opportunity in conducting a clinical study for patients suffering from unusual or specific illnesses [64]. In this point, OOAC becomes an urgent approach for personalized and precision medicine and dentistry in the framework of regenerative therapy. Since OOAC often involves sensors within its device, this cultivates huge potential for personalized medicine in a chairside setting by creating patient-specific drug regimens [64], patient-derived cells engagement [64], or by developing a one-size-fits-all chip for real-time clinical assessment for periodontal disease and caries risk assessment, immunoassay, or oral cancer detection. Especially in relation to alveolar bone damage caused by high prevalence of severe and progressive periodontitis, precise therapeutics strategies are awaiting, and it needs shifting approach from conventional monolayer cell cultures and animal studies into microfluidic alveolar-bone-on-a-chip. The challenges for the next generation of OOAC, including microfluidic alveolar-bone-on-a-chip, include recapitulation of more physiological metabolic phenotypes and patient microbiota to experimentally investigate various gut microbiome dysbiosis, which have been correlated to various chronic diseases in periodontal tissues and, to large extent, oral cavities.

**Executive summary**

**Bone related issues are problematic worldwide**

- Bone-related issues are still problematic worldwide and in dentistry, for example, the issues are reflected by alveolar bone damage and infections found in patients with periodontitis, with high prevalence in numbers and severe progressive conditions. The challenges can be resolved through comprehensive understanding of the complex physiological and pathological processes associated with bones, their responses to different therapeutics strategy, and cell interactions with biomaterials.

**Lack in mimicking physiological, pathological, & regeneration mechanism**

- So far, either existing cell culture model nor pre-clinical animal study have been inadequately mimicking the complex physiological and pathological processes associated with bone microenvironment, functions, and regeneration process in responses to different therapeutic strategies. It brings the consequences for the low success rate of therapeutics strategies in clinical settings.

**Lab-on-chip is crucial for future development**

- The development of microfluidic organ-on-a-chip (OOAC) is crucial to better recapitulate infection site microenvironment and microphysiology within the healthy or diseased tissues and organs, thus OOACs have been applied in various experiments in both fundamental and applied biomedical research, such as in drug discovery, toxicity evaluation, as well as in disease and cancer modeling.

**Advancement in OOAC research**

- Although the numbers are limited, but it was found from this study that OOACs have been used in dentistry and bone tissue engineering to observe various biological processes both in healthy and diseased environments. The results showed that microfluidic OOACs provide better outcomes to resolve complexities during development and translation of a new therapeutics strategy due to the capacity of the OOACs in representing real time microenvironments in the human body.

**Addressing OOAC in dentistry & bone tissue engineering**

- It is expected that dentistry and bone tissue engineering will provide more accurate, precise, faster, and more personalized therapeutics strategies to encounter unpredictable diseases and infections in the future by applying microfluidic OOACs technology, either alone or in combination with other advanced technologies such as stem cells, tissue engineering, or organoids and spheroids technology.
